# Colchicine for the treatment of the spectrum of cardiovascular diseases: current evidence and ongoing perspectives

**DOI:** 10.2459/JCM.0000000000001647

**Published:** 2024-07-19

**Authors:** Massimo Imazio, Cosimo Agrimi, Laura Cescon, Giovanni Panzolli, Valentino Collini, Gianfranco Sinagra

**Affiliations:** a Department of Medicine (DMED), University of Udine; b Cardiothoracic Department, University Hospital Santa Maria della Misericordia, Udine; c Cardiology Specialty School, University of Trieste, Trieste, Italy

**Keywords:** acute coronary syndromes, chronic coronary syndromes, colchicine, pericarditis, prevention, therapy

## Abstract

Colchicine is one of the oldest drugs in medicine. Traditionally used to treat and prevent gouty attacks, it has been introduced into cardiovascular medicine for the treatment and prevention of pericarditis, starting from the positive experience in the treatment and prevention of polyserositis in familial mediterranean fever. Colchicine is a lipophilic drug that enters the cells and is eliminated by glycoprotein P. As granulocytes are lacking in this protein, colchicine is able to concentrate in these cells, exerting a substantial anti-inflammatory action, even with low oral doses. As these cells may trigger acute cardiovascular events, colchicine has been shown to be efficacious and safe to prevent acute coronary syndromes and ischemic stroke with an efficacy comparable to more established treatments, such as antiplatelet agents and statins. On this basis, colchicine seems a promising, efficacious, well tolerated, and cheap option for the prevention of several cardiovascular events, and it may become an additional pillar in the pharmacologic treatment of cardiovascular diseases.

## Introduction

Colchicine is one of the oldest drugs used in medicine. Colchicine has been a herbal remedy for joint pain, first mentioned in Egyptian times in the Ebers Papyrus, an Egyptian medical manuscript written around 1500 BC. The drug has been used for centuries to treat and prevent gouty attacks, but the active ingredient, colchicine, was isolated only in the early 1800s by the French chemists Pierre-Joseph Pelletier and Joseph Bienaimé Caventou, and remains in use today as a purified natural product. The name ‘colchicine’ is derived from the ancient and legendary kingdom of Colchis, from where Jason recovered the Golden Fleece, and where *Colchicum autumnale* plants were widespread.^1,2^ Indeed, the plant is widespread all over the world, and occasional poisonings have been reported in Europe and Italy, as the flowers can be mistaken for saffron.

Colchicine has been studied as an antimitotic agent as it blocks tubulin polymerization and interferes with microtubules function that is essential for mitosis. This is probably the basis for the common scepticism regarding the possible therapeutic use of the drug, and the fear for possible side effects related to this action, such as consequences on fertility and mutagenesis that, however, were never reported at low oral doses in patients with long-term treatments, such as those with familial mediterranean fever (FMF).

The introduction of colchicine into cardiovascular medicine is due to Bayes de Luna. The original hypothesis was that colchicine could prevent recurrences of pericarditis in idiopathic cases, based to its efficacy and safety at low doses for the treatment and prevention of polyserositis attacks of FMF.^2^ Based on this premise, several randomized clinical trials have proved this hypothesis, in the setting of either acute or recurrent pericarditis.^3–7^

Nowadays, we better know the mechanism of action of the drug and its capability to concentrate in granulocytes and have a sustained anti-inflammatory action, even when used at low oral doses (e.g. 0.5 mg/day).^8^ The aim of this article is to review the current evidence supporting the use of this drug, as well as its safety, at low oral doses in several cardiovascular diseases (CVDs), including inflammatory heart diseases, acute and chronic coronary syndromes, arrhythmias triggered by inflammatory mechanisms (e.g. atrial fibrillation after cardiac surgery or ablation), and perhaps myocardial remodelling, a potential application to prevent adverse ventricular remodelling leading to heart failure.

## How colchicine can affect cardiovascular outcomes

**Fig. 1:**
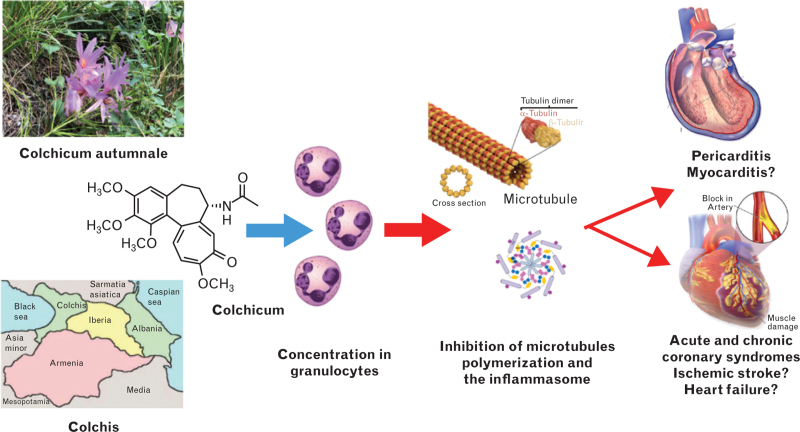
Central illustration. Colchicine mechanisms of action for established cardiovascular indications (pericarditis, acute and chronic coronary syndromes). Additional possible indications include other inflammatory diseases of the heart (myocarditis), atrial fibrillation triggered by inflammation, ventricular remodelling after acute myocardial infarction, and early stages of heart failure (see additional explanations in the text).

Colchicine is a lipophilic drug derived from the plant of *C. autumnale* (Fig. 1). The name is after Colchis, an ancient region in the Black Sea, where these plants were widespread.^1^ Indeed, the plant is ubiquitous and the flowers are rather similar to saffron flowers.

Colchicine is absorbed by the jejunum and ileum. Bioavailability is variable (mean 45%); however, peak serum concentrations are usually reached within 0.5–3 h of oral administration, and decline over the next 2 h, but then rise again because of enterohepatic recycling. Colchicine has an elimination half-life of 27–31 h. Thus, once ceased, its biological effects on leukocytes decline after 24–48 h. Colchicine is partially metabolized in the liver by de-acetylation with a half-life elimination of 12–30 min and is predominantly eliminated by the kidneys and in the bile.^9–11^ Decreased clearance through either of these two pathways may increase the risk of drug accumulation. Two major interactions of colchicine with specific proteins modulate its pharmacokinetics beyond tubulin: cytochrome P450 3A4 (CYP3A4) and P-glycoprotein. Cytochrome P450 3A4 metabolizes colchicine in the liver. P-glycoprotein is an ATP-dependent phosphor-glycoprotein located in the cell membrane and responsible for the excretion of the drug in the intestine, liver, kidney, and blood–brain barrier.^11,12^

The drug freely enters the cells and blocks tubulin polymerization that is central for the assembly of microtubules, a key element of the cellular cytoskeleton. Microtubules are essential for the motility and function of these cells, and the assembly of a complex of cytosolic proteins, the inflammasome, that is activated in inflammatory states. Moreover, microtubules are responsible for the expression of adhesion proteins, such as the selectins, that are responsible for the interactions between different cells (e.g. endothelial cells, platelets, inflammatory cells).^1,13^

The drug is eliminated from cells by P-glycoprotein; such protein is not present on granulocytes, where the drug is able to concentrate even at low oral doses (e.g. 0.5 mg/day), exerting a sustained anti-inflammatory action linked to the inhibition of microtubules function, and affecting several functions of these cells (e.g. chemiotaxis, degranulation, phagocytosis), as well as the assembly of the inflammasome, thus reducing the release of pro-inflammatory cytokines released through the activation of this complex (e.g. interleukin 1, IL-1).^1,8^

Therefore, colchicine directly inhibits neutrophil chemotaxis and activity in response to vascular injury, indirectly reduces the production of active interleukin IL-1β via inhibitory effects on the inflammasome,^5^ and reduces neutrophil–platelet aggregates, which may accumulate in the microvascular beds, for example, during acute myocardial infarction (MI) or contributing to myocardial injury after percutaneous coronary intervention.^9^

## First indication of colchicine for cardiovascular diseases: pericarditis

In cardiovascular medicine, the first indication for colchicine has been the treatment and prevention of idiopathic recurrent pericarditis. Based on the efficacy and safety of the drug to prevent attacks of serositis in FMF, Bayes de Luna *et al.* proposed the use of the drug also in isolated cases with idiopathic recurrent pericarditis. They reported the anecdotal treatment of three cases in a research letter in the *Lancet* in 1987.^2^ This preliminary experience was later confirmed in case series, but only later, several clinical trials (Table 1) clearly demonstrated the efficacy of colchicine to halve the recurrence rate of pericarditis in the majority of clinical trials, either in patients with a first episode of pericarditis^10,11^ or in those with recurrences,^12–14^ but a smaller Spanish study was probably not adequately powered.^15^ In this study, in acute pericarditis (first episode), only 110 patients were enrolled (59 vs. 51 patients) and the follow-up was completed in only 92% of cases. With a study hypothesis that colchicine could halve the recurrence rate of 30%, a minimal sample size of 240 cases was needed (alpha 0.05, power 80%). Currently, the best therapeutic scheme is based on a weight-adjusted dose (0.5 mg once daily for patients <70 kg or 0.5 mg twice daily), without a loading dose, and with a duration of treatment of 3 months for the first episode of pericarditis, and 6 months for recurrent pericarditis.^11,14^ Colchicine is always added on top of standard anti-inflammatory therapy based on NSAID or corticosteroids for those who are not responsive to NSAID or with intolerance/contraindications to these drugs. The most common side effect is gastrointestinal intolerance reported in 7–10% of cases.^3,10–14^

**Table 1:** Major clinical trials on the use of colchicine for pericarditis

Study	Study design	Colchicine dose and duration	Clinical setting	Patients number	Main results
COPE trial^10^ (2005)	Randomized trial (open label)	Colchicine, 1 mg on first day, followed by 0.5 mg daily (if <70 kg) or 1 mg twice daily followed by 0.5 mg twice daily (if ≥70 kg), for 3 months	Acute pericarditis	120	Reduction of recurrent pericarditis (11 vs. 32%, *P* < 0.01, NNT 5) and symptoms persistence at 72 h (12 vs. 37%, *P* < 0.01)
CORE trial^12^ (2005)	Randomized trial (open label)	Colchicine, 1 mg on first day, followed by 0.5 mg daily (if <70 kg) or 1 mg twice daily followed by 0.5 mg twice daily (if ≥70 kg), for 6 months	First recurrence of pericarditis	84	Reduction of recurrent pericarditis (24 vs. 51%, *P* = 0.02, NNT 4) and symptoms persistence at 72 h (10 vs. 31%, *P* = 0.03)
CORP trial^13^ (2011)	Double-blind RCT	Colchicine, 1 mg on first day followed by 0.5 mg daily (if <70 kg) or 1 mg twice daily followed by 0.5 mg twice daily (if ≥70 kg), for 6 months	First recurrence of pericarditis	120	Reduction of recurrent pericarditis (24 vs. 55%, *P* < 0.01) and symptoms persistence at 72 h (23 vs. 53%, *P* < 0.01)
ICAP trial^11^ (2013)	Double-blind RCT	Colchicine, 0.5 mg daily (if <70 kg) or 0.5 mg twice daily (if ≥70 kg), for 3 months	Acute pericarditis	240	Reduction of recurrent or incessant pericarditis (17 vs. 37%, *P* < 0.01, NNT 4) and symptoms persistence at 72 h (19 vs. 40%, *P* < 0.01)
CORP-2 trial^14^ (2014)	Double-blind RCT	Colchicine, 0.5 mg daily (if <70 kg) or 0.5 mg twice daily (if ≥70 kg), for 6 months	Recurrent pericarditis (second or subsequent recurrence)	240	Reduction of recurrent pericarditis (22 vs. 42%, *P* < 0.01, NNT 5)
Sambola *et al.*^15^ (2019)	Randomized trial (open label)	Colchicine, 0.5 mg twice daily (if <70 kg) or 1 mg twice daily (if ≥70 kg), for 3 months	Acute pericarditis	110	Failure to reduce recurrent pericarditis (13 vs. 8%, *P* = NS)
Finkelstein *et al.*^16^ (2002)	Randomized trial (open label)	Colchicine, 1.5 mg daily from the third postoperative day, for 1 month	Postpericardiotomy syndrome following cardiac surgery	163	Failure to reduce postpericardiotomy syndrome (11 vs. 22%, *P* = 0.135)
COPPS trial^17^ (2010)	Double-blind RCT	Colchicine, 1 mg on the third postoperative day followed by 0.5 mg daily (if <70 kg) or 1 mg twice daily followed by 0.5 mg twice daily (if ≥70 kg), for 1 month	Postpericardiotomy syndrome following cardiac surgery	360	Reduction of postpericardiotomy syndrome (9 vs. 21%, *P* < 0.01)
COPPS-2^18^ (2014)	Double-blind RCT	Colchicine from 48 to 72 h before surgery, 0.5 mg daily (if <70 kg) or 0.5 mg twice daily (if ≥70 kg), for 1 month	Postpericardiotomy syndrome following cardiac surgery	360	Reduction of postpericardiotomy syndrome (19 vs. 29%, *P* < 0.01) although it did not reduce occurrence of postoperative AF (34 vs. 42%, *P* = NS) or pericardial/pleural effusion (57 vs. 59%, *P* = NS)
Meurin *et al.*^19^ (2015)	Double-blind RCT	Colchicine, 1 mg daily for 2 weeks	Pericardial effusion following cardiac surgery	197	Failure to reduce effusion volume on a 0–4 scale (−1.1 ± 1.3 vs. −1.3 ± 1.3 grades) or late cardiac tamponade (7 vs. 6%, *P* = NS)

AF, atrial fibrillation; NNT, number needed to treat; RCT, randomized controlled trial.

Colchicine was also efficacious for the prevention of the postpericardiotomy syndrome.^16–18^ On the contrary, colchicine was not efficacious in the setting of noninflammatory postoperative pericardial effusions.^19^

## Colchicine as first successful anti-inflammatory drug for atherosclerotic plaques

Colchicine has been the first really successful anti-inflammatory drug for the secondary prevention of cardiovascular events aside from the experience of the CANTOS study on canakinumab (Table 2),^20^ but with the additional major advantage of being a cheaper option for the anti-inflammatory therapy of atherosclerosis. The preliminary key finding was the observation that colchicine decreased high sensitivity levels of C-reactive protein after 30 days of treatment in patients with stable coronary disease on optimal medical therapy, suggesting that colchicine had anti-inflammatory effects over statin and antiplatelet therapy.^21^ The important role on inflammation in the vulnerability of cardiovascular plaques is already known from other studies.^22–24^

**Table 2:** Major clinical trials on the use of colchicine for acute and chronic coronary syndromes

Study	Study design	Colchicine dose and duration	Clinical setting	Patients number	Main results
LoDoCo trial^25^ (2013)	Randomized trial (observer blinded)	Colchicine 0.5 mg daily for a median of 36 months plus statins and standard secondary prevention drugs	Stable coronary artery disease	532	Reduction of cardiovascular events (ACS, out-of-hospital cardiac arrest, noncardioembolic ischemic stroke): 5.3 vs. 16% (HR 0.33, 95% CI 0.18–0.59)
LoDoCo trial^28^ (2020)	Double-blind RCT	Colchicine 0.5 mg daily vs. placebo	Stable coronary artery disease	5522	Reduction of CV death, myocardial infarction, ischemic stroke, or ischemia-driven coronary revascularization: 6.8 vs. 9.6% (HR 0.69, 95% CI 0.57–0.83)
COLCOT trial^29^ (2019)	Double-blind RCT	Colchicine 0.5 mg daily for a median of 20 months	Recent myocardial infarction (<1 month)	4745	Reduction of cardiovascular events (composite of cardiovascular death, cardiac arrest, myocardial infarction, stroke or urgent hospitalizations for angina): 5.5 vs. 7.1% (HR 0.77, 95% CI 0.61–0.96)
COPS trial^31^ (2020)	Double-blind RCT	Colchicine 0.5 mg twice daily for the first month, then 0.5 mg daily	Acute coronary syndromes	795	The primary outcome of all-cause mortality, ACS, ischemia-driven (unplanned) urgent revascularization, and noncardioembolic ischemic stroke did not differ between colchicine (*n* = 396) and placebo (*n* = 399): 24 vs. 38 events (*P* = 0.09)

ACS, acute coronary syndromes; CV, cardiovascular; HR, hazard ratio; RCT, randomized controlled trial.

The first clinical trial (LoDoCo) conducted on clinical outcomes evaluated the efficacy of low-dose colchicine (0.5 mg/day) to reduce a composite endpoint of cardiovascular events in 532 patients with stable coronary artery disease (acute coronary syndromes, fatal out-of-hospital cardiac arrest, noncardioembolic ischemic stroke). In this randomized, open-label trial, colchicine reduced the combined endpoint [hazard ratio 0.33, 95% confidence interval (CI) 0.18–0.59], regardless of baseline C-reactive protein (CRP) levels, on top of optimal medical therapy including antiplatelet drugs and statins.^25^ Subsequently observational studies in gouty patients demonstrated that colchicine significantly reduced the risk of cardiovascular events, including MI, transient ischemic attack, stroke [odds ratio (OR) 0.51, 95% CI 0.30–0.88], and all-cause mortality (hazard ratio 0.27, 95% CI 0.17–0.43); all outcomes were prespecified as primary or secondary endpoints of the studies.^26,27^

However, the major clinical trial that investigated the effect of colchicine on stable coronary artery disease (CAD) is the LoDoCo2 trial, in which a total of 5522 patients with stable ischemic heart disease, who were tolerant to an open-label treatment with colchicine 0.5 mg/daily, were randomized to receive colchicine 0.5 mg/daily or placebo.^28^ As far as ACS are concerned, one of the most relevant trials is the Colchicine Cardiovascular Outcomes Trial (COLCOT) that evaluated the efficacy and safety of colchicine in patients with an acute coronary syndrome designed similarly to LoDoCo2.^29^

In the LoDoCo2 trial, after a median follow-up of 29 months, colchicine significantly reduced the primary endpoint (cardiovascular death, spontaneous MI, ischemic stroke, or ischemia-driven coronary revascularization) compared with the placebo group (hazard ratio, 0.69; 95% CI 0.57–0.83; *P* < 0.001) without significant side effects.^28^

In the COLCOT, patients with a recent (<1 month) MI were randomized to colchicine 0.5 mg daily or placebo and followed up for 4 years. In this trial, colchicine reduced the incidence of the composite of cardiovascular death, cardiac arrest, MI, stroke or urgent hospitalizations for angina (hazard ratio 0.77, 95% CI 0.61–0.96). The outcome was mainly driven by a reduction in the incidence of stroke and urgent revascularization for angina.^29^ A subsequent substudy of COLCOT suggested that the efficacy improved with an earlier treatment when colchicine was initiated within 3 days of the onset of MI (hazard ratio 0.52, 95% CI 0.32–0.84).^30^ In the COLCOT trial, colchicine use was associated with a low but increased incidence of hospitalization for (nonfatal) pneumonia (0.9 vs. 0.4%, *P* = 0.03).^29^ On the other hand, other studies did not show significant results in terms of reduction of major adverse cardiovascular events (MACE) in patients with ACS treated with colchicines.^31,32^ However, the 2023 European Society of Cardiology (ESC) guidelines on ACS suggest that the use of low-dose colchicine may be considered when optimal medical therapy is not sufficient.^33^

Several systematic reviews have summarized the available evidence on colchicine for acute and chronic coronary syndromes. A meta-analysis pooling data from all of the major trials on colchicine and CAD (11 594 patients) showed a reduction of 32% of the composite of cardiovascular mortality in patients treated with colchicine.^34^ Overall, including the five major clinical trials (Table 2), colchicine reduced the risk for the primary endpoint (MACE, MI, stroke, or cardiovascular death) by 25% [relative risk (RR) 0.75, 95% CI 0.61–0.92; *P* = 0.005], MI by 22% (RR 0.78, 95% CI 0.64–0.94; *P* = 0.010), stroke by 46% (RR 0.54, 95% CI 0.34–0.86; *P* = 0.009), and coronary revascularization by 23% (RR 0.77, 95% CI 0.66–0.90; *P* < 0.001). Colchicine did not affect all-cause death (RR 1.08, 95% CI 0.71–1.62; *P* = 0.73), with a trend for a lower incidence of cardiovascular death (RR 0.82, 95% CI 0.55–1.23; *P* = 0.34), but an apparent trend towards a higher incidence of noncardiovascular death (RR 1.38, 95% CI 0.99–1.92; *P* = 0.060)^35^ that gave rise to several criticisms and scepticism on the use of colchicine for secondary cardiovascular prevention in ischemic heart disease.

Moreover, in the ACS setting, colchicine could significantly reduce MMP-9, NOX2, and TGF-β1 levels in stable STEMI patients. These biomarkers are tightly connected with cardiac remodelling. Colchicine improves right ventricular function and t-tubule architecture, although reducing microtubule density and junctophilin-2 expression. Therefore, colchicine could be a potential agent in STEMI patients and prevent cardiac remodelling events.^36^

### Colchicine in preventing restenosis after coronary angioplasty

Percutaneous coronary intervention (PCI) has been considered as one important indication for an anti-inflammatory drug such as colchicine to prevent progression of atherosclerosis and restenosis. In this setting, colchicine might play an important role, especially reducing the inflammation related to the procedure, resulting in a possible decrease in post-PCI cardiovascular events, such as in-stent restenosis. PCI-related myocardial injury may be partly because of wire injury, microdissections at the site of balloon inflations, and vascular trauma due to high-pressure balloon inflations. PCI-related myocardial injury may also result from mechanical events, such as distal microembolization and side branch occlusion from plaque shift. A proinflammatory state during PCI may lead to endothelial dysfunction and leukocyte–platelet aggregates in distal beds, which, in turn, can limit the ability of the coronary microvasculature to accommodate atherothrombotic debris. Despite the use of contemporary techniques, devices, and pharmacology, systemic inflammation at the time of PCI is associated with adverse events, including cardiac death, stent thrombosis, and target lesion revascularization, as early as 30 days post-PCI.

Nevertheless, preprocedural administration of colchicine did not lower the risk of PCI-related myocardial injury, PCI-related MI, or MACE at 30 days when compared with placebo but did significantly attenuate the increase in IL-6 and hsCRP concentrations 22–24 h post-PCI when compared with placebo. Finally, PCI was not associated with increased levels of IL-1β, suggesting that IL-1β may not be an appropriate target for vascular injury and inflammation in this setting. The use of colchicine, although theoretically promising, was ineffective in preventing restenosis after coronary angioplasty. Although the use of antineoplastic and antimitogenic agents in this application merits further consideration, therapy with higher doses and more potent agents will be limited to some degree by adverse effects related to such regimens. Nevertheless, colchicine prevented an acute rise in inflammatory biomarkers in an acute setting, and the lack of benefit of colchicine on PCI-related myocardial injury may be attributable to the pharmacodynamics of colchicine, including too short a time period for colchicine administration pre-PCI and/or insufficient doses, particularly in the setting of acute coronary syndrome and mechanical intra-procedural complications, plaque shift, and distal emboli.^9^ Therefore, more studies are needed in the PCI setting, focusing on delivery systems to allow local application of colchicine, and improving drug dosing, compliance, and reduction of side effects.^37^

### New possible indications beyond pericarditis and ischemic heart diseases

Colchicine has three main well recognized mechanisms of action: nonselective inhibition of the inflammasome, inhibition of neutrophil functions by concentration in these cells and inhibition of microtubules function, and interference with selectins and interaction of platelets and neutrophils. New potential indications include the prevention of stroke, the treatment of myocarditis and heart failure to prevent myocardial remodelling, and the prevention of atrial fibrillation mediated by inflammatory mechanisms.

### Ischemic stroke prevention

As pointed out, if we include the five major clinical trials that evaluated colchicine for the secondary prevention of cardiovascular events, colchicine reduced the risk of stroke by 46% (RR 0.54, 95% CI 0.34–0.86; *P* = 0.009).^35^ It is not clear if this effect is only due to the anti-inflammatory action on atherosclerotic plaques or other mechanisms (e.g. prevention of atrial fibrillation triggered by inflammation). There are different ongoing trials evaluating the effects of colchicine in patients with previous stroke. The open-label Colchicine for prevention of vascular inflammation in noncardio embolic stroke trial (CONVINCE) will examine its effects in patients with a recent noncardioembolic transient ischemic attack or ischemic stroke, with a plan to enlist 2623 patients (ClinicalTrials.gov ID NCT02898610). The Efficacy of Colchicine in Preventing Recurrent Stroke in the Patients With Acute Atherothrombotic Ischemic Stroke During Hospitalization trial (COLCHIDA) is a prospective, randomized, open-label, controlled study that aims to examine the efficacy of low-dose colchicine in preventing recurrent stroke in patients with previous ischemic stroke during hospitalization (ClinicalTrials.gov Identifier: NCT06102720). An additional interesting trial is the Colchicine in High-risk Patients With Acute Minor-to-moderate Ischemic Stroke or Transient Ischemic Attack trial (CHANCE-3), a multicentre, randomized, double-blind, placebo-controlled study that will investigate the efficacy of colchicine in this setting.^38^

### Myocarditis and heart failure

Colchicine is an established treatment for pericarditis not only to treat the acute episode but also to prevent recurrences. The effect is related to its capability to concentrate in granulocytes and to inhibit the inflammasome activation with the generation of pro-inflammatory cytokines, first of all, IL-1. Myocarditis is another inflammatory condition in which colchicine could be useful in the early stages of the disease. In a recently published animal model of coxsackievirus 3 (CVB3)-induced myocarditis in mice, colchicine improved left ventricular (LV) function, and decreased the release of troponin as well as cardiac and splenic NLRP3 inflammasome activity, without exacerbation of the viral load.^24^ The use of colchicine in human myocarditis remains an interesting hypothesis to be further explored in specifically designed clinical studies. At present, a retrospective study has demonstrated the safety and efficacy of colchicine on top of standard anti-inflammatory therapies in patients with myopericarditis.^4^

Colchicine interferes with several steps in the inflammatory process, including NLRP3 inflammasome suppression responsible for IL-1 and IL-6, and influences CRP production.

An initial study was designed to test the efficacy of a 6-month course of anti-inflammatory treatment with colchicine in improving functional status of patients with stable chronic heart failure. In this study, although effective in reducing inflammation biomarker levels, colchicine did not affect patient functional status [New York Heart Association (NYHA) class and objective treadmill exercise tolerance] or the likelihood of death or hospital stay for heart failure.^39^ Ongoing research is now focused to assess the efficacy of colchicine to reduce systemic low-grade inflammation in patients with heart failure.

Systemic low-grade inflammation is one of the fundamental underlining mechanisms of heart failure with preserved ejection fraction (HFpEF) involved in cardiomyocyte stiffening and myocardial fibrosis. Increased levels of inflammatory biomarkers including CRP, interleukin (IL)-1 and IL-6, and soluble suppression of tumorigenicity 2 (sST2) have been associated with disease progression and adverse outcomes in these patients.

Among others, sST2 is acknowledged as a prognostic biomarker of heart failure and improves risk stratification for death when added to N-terminal pro-B-type natriuretic peptide (NT-proBNP). Anti-inflammatory therapies in HFpEF are largely understudied.

In an ongoing study, the effects of the administration of colchicine will be assessed in patients with HFpEF. Inflammatory markers such as sST2 and hsCRP will be evaluated at baseline and at 12-week follow-up. The secondary objective of the study will be to determine if treatment with colchicine influences N-terminal pro-B-type natriuretic peptide levels, LV diastolic function and remodelling, right ventricular systolic function and left atrial volumetric characteristics.^40^

There is another interesting ongoing trial, the COLpEF trial. The COLpEF will be a randomized, blinded, multiple-dose, placebo-controlled trial. The primary endpoint will be the change between baseline and 6 months in high-sensitivity CRP. Secondary objectives will be the change in NT-pro brain natriuretic peptide and high-sensitivity troponin; and coronary flow reserve, assessed using adenosine-based PET imaging. Tertiary objectives will be the change in NYHA class and 6-minute walk test.^41^

New studies are needed to address the potentiality of colchicine to prevent ventricular remodelling through its anti-inflammatory properties.

### Atrial fibrillation

Numerous animal studies have demonstrated the important role of inflammation in atrial fibrillation and the potential benefits of colchicine use. Colchicine may prevent atrial fibrillation with different anti-inflammatory mechanisms: inhibition of IL-6 release and consequent fibrosis; activation of the PI3K/AKT/eNOS signalling pathway, which may reverse atrial remodelling; and reduction of NLRP3 inflammasome activity.^42–44^ A recent meta-analysis^45^ evaluated the use of colchicine in preventing atrial fibrillation in three different settings: postoperative atrial fibrillation (POAF) after cardiac surgery, atrial fibrillation recurrence following pulmonary vein ablation/isolation, and atrial fibrillation in patients with CAD. The meta-analysis found that colchicine is beneficial in decreasing the incidence of POAF, with a relative risk of 0.7 (95% CI 0.58–0.84). In particular, the COPPS trial and COCS trial demonstrate the beneficial effect of colchicine in POAF, whereas the other major trials had a neutral result. The COPPS POAF substudy included 336 patients of the COPPS trial who were in sinus rhythm before the surgery. They received placebo or colchicine 1.0 mg twice daily starting on postoperative day 3 followed by a maintenance dose of 0.5 mg twice daily for 1 month in patients 70 kg (halved doses if <70 kg or in case of intolerance to the highest dose). At 1 month, patients on colchicine had a reduced incidence of POAF (12 vs. 22%, respectively; *P* = 0.021; relative risk reduction, 45%).^46^ The COCS trial is a double-blind randomized placebo-controlled trial that included 267 patients. Patients received colchicine on the day before the surgery and on postoperative days 2, 3, 4, and 5. The rhythm was controlled after the operation and until discharge from the hospital. POAF was observed in 18.6% patients of the colchicine group vs. 30.7% of the control patients (OR 0.515; 95% Cl 0.281–0.943; *P* = 0.029).^47^ The same meta-analysis evaluated patients who underwent pulmonary vein isolation/ablation, including three important studies.^48–50^ In patients who underwent PVI/ablation, colchicine was beneficial in decreasing atrial fibrillation recurrence over 3 months with an RR of 0.57 (95% CI 0.39–0.83) and over 12 months of follow-up with an RR of 0.58 (95% CI 0.42–0.80). However, in the meta-analysis, colchicine had no significant benefit in decreasing the incidence of atrial fibrillation in patients with stable/unstable coronary artery disease (hazard ratio 0.86, 95% CI 0.69–1.06). The same lack of efficacy of colchicine to prevent atrial fibrillation in the setting of ischemic heart disease was demonstrated in sub-analyses of the COLCOT and LoDoCo2 trials.

The COP-AF trial is a recent important trial, not included in the previous meta-analysis that investigated patients undergoing major noncardiac thoracic surgery. In patients undergoing major noncardiac thoracic surgery, administration of colchicine did not significantly reduce the incidence of clinically important atrial fibrillation but increased the risk of mostly benign noninfectious diarrhoea (Table 3).^51^

**Table 3:** Major clinical trials on the use of colchicine for the prevention of atrial fibrillation

Study	Study design	Colchicine dose and duration	Clinical setting	Patients number	Main results
COPPS-AF^46^ (2011)	Double-blind RCT	Colchicine from 48 to 72 h before surgery, 0.5 mg daily (if <70 kg) or 0.5 mg twice daily (if ≥70 kg), for 1 month	Cardiac surgery	360	No significant difference
COCS trial^47^ (2022)	Double-blind randomized placebo-controlled trial	1 mg dose of colchicine or placebo 24 h before surgery and on days 2, 3, 4, and 5	Cardiac surgery	240	The colchicine group developed POAF in 18.6% of cases, the placebo group in 30.7% of cases, confirming the beneficial effect of colchicine in preventing POAF after cardiac surgery
COP-AF trial^51^ (2023)	Randomized trial	0.5 mg twice daily or placebo, starting within 4 h before surgery and continuing for 10 days	Major noncardiac thoracic surgery	3209	Colchicine did not significantly reduce the incidence of clinically important atrial fibrillation
Deftereos *et al.*^48^ (2012)	Randomized trial	0.5 mg twice daily from the day of ablation	Postpulmonary vein isolation	161	In the 3-month follow-up, 33.5% of patients in the placebo group and 16% of patients in the colchicine group had a recurrence of AF
Egami *et al.*^49^ (2015)		0.5 mg/day for 2 weeks after AF ablation	Postablation patients in relation to left atrial epicardial adipose tissue	122	Colchicine might reduce AF recurrence in patients with larger LA volumes (incidence of AF recurrence at 12 was 10.5 vs. 34.2%, *P* = 0.06)
Deftereos *et al.*^50^ (2014)	Randomized trial	0.5 mg twice daily or a placebo for 3 months from the day of the ablation procedure	Postpulmonary vein ablation	206	In the 12-month follow-up, the colchicine group had AF recurrence of 31.1% (32/103) and the control group had 49.5% (51/103)

AF, atrial fibrillation; CV, cardiovascular; LA, left atrium; POAF, postoperative atrial fibrillation; RCT, randomized controlled trial.

There is a need for further research to confirm the beneficial effects of colchicine against atrial fibrillation. There is an interesting ongoing trial, the IMPROVE-PVI Pilot trial (ClinicalTrials.gov Identifier: NCT04160117), that will evaluate the impact of a 10-day treatment with colchicine to improve patient relevant outcomes after catheter ablation for atrial fibrillation.

## Food and Drug Administration approval for colchicine in United States of America

The 2021 ESC guidelines on cardiovascular prevention introduced a recommendation for the use of anti-inflammatory therapy with colchicine. According to these guidelines, low-dose colchicine (0.5 mg one daily) may be considered in secondary prevention of CVD, particularly if other risk factors are insufficiently controlled or if recurrent CVD events occur under optimal therapy.^52^ The level of evidence to support this IIb recommendation is A, suggesting a scepticism of the guidelines task force to encourage this type of preventive treatment, although clinical trials such as LoDoCo2 and COLCOT have provided evidence of a 25–30% reduction of MACE, regardless of the levels of CRP, with an efficacy comparable to statins. Indeed, in ESC guidelines, data derived by multiple randomized controlled trials or metanalyses support a level of evidence (LOE) A as in this case, and it is usually associated with a recommendation of class I or, at least, IIa, if the overall evidence is in favour of a treatment as for colchicine in this condition.

On the contrary, based on current available evidence, in June 2023, the US Food and Drug Administration approved the use of low-dose colchicine to reduce the risk of MI, stroke, coronary revascularization, and cardiovascular death in adult patients with established atherosclerotic disease or with multiple risk factors for CVD. Approval for secondary prevention of ischemic heart diseases is still pending in Europe.

## Side effects and safety profile: why prescription is still limited in Europe

There is a common scepticism on the safety of colchicine. These concerns probably are related to the well known antimitotic effect of colchicine raising suspicions on possible side effects. These doubts have been fostered by the evidence of an increased incidence of hospitalization for (nonfatal) pneumonia in the COLCOT trial, as well as evidence of a trend towards a higher incidence of noncardiovascular death reported in trials and systematic reviews.

A recent article has clearly addressed that the use of low doses of colchicine is not associated with increased mortality.^53^ In the LoDoCo2 trial, after a median 28.6 months of follow-up, 133 out of 5522 participants (2.4%) died. Cardiovascular deaths were similar in patients with or without colchicine. Recorded noncardiovascular deaths included: cancer, end-stage pulmonary disease, infections, dementia, and multiple organ failure, all equally distributed in patients treated with or without colchicine. Multivariable analysis demonstrated age older than 65 years was the only independent baseline characteristic associated with noncardiovascular death (hazard ratio 3.65; 95% CI 2.06–6.47). On this basis, colchicine was not associated with increased mortality. Most deaths were related to noncardiovascular causes, underlying the importance of comorbidities in these patients.^53^

A recently published meta-analysis also pointed out clearly that the beneficial effects of the drugs can be achieved with low doses (0.5 mg/day). Low-dose colchicine significantly reduced MACE (RR 0.51; 95% CI 0.32–0.83), recurrent MI (RR 0.56; 95% CI 0.35–0.89), stroke (RR 0.48; 95% CI 0.23–1.00), and hospitalization (RR 0.44; 95% CI 0.22–0.85), whereas high and loading doses significantly increased gastrointestinal adverse events (RR 2.84; 95% CI 1.26–6.24) and discontinuation (RR 2.73; 95% CI 1.07–6.93).^35^

Although the therapeutic window of colchicine is narrow, when used at low doses, colchicine is well tolerated and efficacious for treatment, while loading doses and higher doses only increase the risk of side effects, intolerance, and drug withdrawal. It is noteworthy that data accrued over the last 50 years strongly suggest that the biologic effects of long-term colchicine do not increase the risk of cancer, sepsis, cytopenia, or myotoxicity.^5^

## Conclusion and future perspectives

In a recently published review by Ridker *et al.* that summarizes current opinion on the use of colchicine for secondary prevention of ischemic heart diseases, it is remarked that low-dose (0.5 mg/day) colchicine reduces cardiovascular event rates by 25–30% in patients with coronary atherosclerosis.^54^ US experts suggest that low-dose colchicine should be considered for patients with stable ischemic heart disease who, despite guideline-directed therapy, have high-sensitivity CRP concentrations greater than 2 mg/l, but it should be avoided in patients with renal or hepatic impairment or those concomitantly taking CYP3A4/P-glycoprotein inhibitors.^54^ However, the efficacy of colchicine has been demonstrated in the LoDoCo and COLCOT trials regardless of baseline levels of CRP, and on top of standard medical therapy, including antiplatelet agents and statins without significant interferences reported with low doses (0.5 mg/day).

In conclusion, colchicine seems a promising and safe option for cardiovascular treatment and prevention of established inflammatory diseases, such as pericarditis, and a new option to be tested for myocarditis. Colchicine is also a very promising and cheap therapeutic option for the secondary prevention of cardiovascular events in patients with atherosclerosis,^55^ probably not only confined to coronary atherosclerosis (ischemic stroke and peripheral atherosclerotic artery disease) but also a potential option for ventricular remodelling and early phases of heart failure. Ongoing clinical trials are warranted to test these new potential indications.

### Conflicts of interest

Ther are no conflicts of interest.
